# The Best Under Stress: An Analysis of Breast Tissue Expander Response to External Forces

**DOI:** 10.1093/asjof/ojad018

**Published:** 2023-02-20

**Authors:** Daniel Najafali, Farrah C Liu, Karanvir Raman, Bhagvat Maheta, Golddy Milagros Saldana, Lucas Heldman, Priscila Cevallos, Rahim Nazerali

## Abstract

**Background:**

The use of breast tissue expanders (TEs) in breast reconstruction is accompanied by undesired changes to the chest wall and lateral plane. Breast TEs are designed to create a naturally formed breast pocket by capitalizing on the ductile response of skin tissue; however, in practice, the use of expanders is accompanied by undesired changes to the chest wall and lateral plane.

**Objectives:**

The authors of this study compared 3 comparably sized and commercially available breast TEs to assess the mechanical profile and functionality of each design.

**Methods:**

Authors compared MENTOR Artoura PLUS Smooth (Irvine, CA), Allergan 133 Smooth (Irvine, CA), and Sientra AlloX2 Smooth (Santa Barbara, CA) filled to 100% of their label volume. The mechanical profile of TEs was assessed via vertical compression. Dimensions were recorded at baseline and percent changes were calculated at each compressive load (5-35 lbf intervals of 5 lbf).

**Results:**

Base width and projection were recorded at compressive loads of 10, 20, and 35 lbs. For percent changes of base width, MENTOR had 0.98%, 2.09%, 3.84%; Allergan 4.21%, 9.15%, 15.52%; and Sientra 4.72%, 10.19%, 19.15%. For percent changes of projection, MENTOR had −19.06%, −25.44%, −30.88%, Allergan −35.53%, −42.90%, −50.09%, and Sientra −29.64%, −37.68%, −44.69%. For percent change of height, MENTOR had 1.44%, 2.62%, 4.27%, Allergan 10.26%, 16.49%, 22.97%, and Sientra 6.99%, 11.93%, 16.90%. MENTOR's TE had the most pronounced lower pole with volume expansion.

**Conclusions:**

The MENTOR TE demonstrated the least lateral deformation and projection loss across the range of compressive loads, as well as the highest force resistance compared with the other models.

**Level of Evidence: 3:**

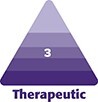

While there is an ever-increasing complexity in multimodal breast cancer management, staged alloplastic breast reconstruction remains the primary strategy for plastic surgeons in the United States.^1^ This approach expands the available options in reconstructive outcomes, especially for patients who require adjuvant radiation therapy.^1-3^ A 2-stage reconstruction utilizes a breast tissue expander (TE) at the time of mastectomy for temporary aesthetic reconstruction and develops a breast pocket to maximize the native breast skin envelope. Following in-clinic expansion to a desired volume and confirmation of cancer eradication, a secondary surgical intervention is performed to replace the TE for the final permanent implant.^1,2^

The ideal TE creates an accurate and predictable breast pocket with shape, size, and positioning reflective of the final implant. It is crucial to maintain breast base width during the expansion process to achieve desired aesthetic outcomes and ensure appropriate matching of the breast width to the TE and implant width under direct pressure forces of the mastectomy skin flap. Furthermore, to obtain the natural teardrop shape of a breast, expansive preference should be focused on the lower pole to ensure forward projection.^4^

Unfortunately, given the dynamic and temporary properties of the device, the expansive capacity is not always readily predictable nor in line with desired outcomes. The most common unwanted effects include the lateral distortion of the base and reduced forward projection of the TE due to restrictions by anteroposterior forces of the chest wall and soft tissues.^4^ Additional factors outside of the reconstructive surgeon's control, such as mastectomy over dissection, loss of definition of the inframammary fold, and adjuvant radiotherapy, also have an impact on the final outcome.^5,6^ Radiation can induce fibrosis of the pectoralis muscle which can further distort the TE superolaterally in subpectoral reconstructions. Techniques such as over-filling the TE by upwards of 30% in volume and second-stage procedures with additional capsule manipulation during the second stage are used to create an ideal breast pocket.^4,7,8^

Commercial manufacturers have responded to these concerns via several design iterations of TEs. However, this poses an additional challenge to surgeons, as the variability and predictability in expansive behavior are unknown. In response to this knowledge deficit, the authors of this paper set out to characterize the mechanical properties of 3 commonly available TEs by utilizing benchtop testing through a vertical compression test, applying variable pressures in the anteroposterior plane, thus scrutinizing the TEs’ propensity for lateral distortion and ability to maintain lower pole projection. The results of these studies are meant to elucidate TE behavior based on compression to surgeons in achieving desired outcomes and selecting an appropriate device for 2-stage reconstruction.

## METHODS

### Tissue Expander Devices

This study examined commercially available breast TEs from 3 separate companies (MENTOR, Irvine, CA; Allergan, Irvine, CA; and Sientra, Santa Barbara, CA). The design of each TE was evaluated for its mechanical profile and overall functionality. Vertical compression testing was conducted on the following TEs summarized in Supplemental Table 1: MENTOR Artoura PLUS Smooth High Profile 600 cc (Product code: SDC-140H), Allergan 133 Smooth Moderate High Extra Projection 600 cc (Product code: 133S-MX-14-T), and Sientra AlloX2 Smooth Full Height 575 cc (Product code: AlloX2-FH-14SE).

### Mechanical Vertical Compression Testing

**Figure 1.**
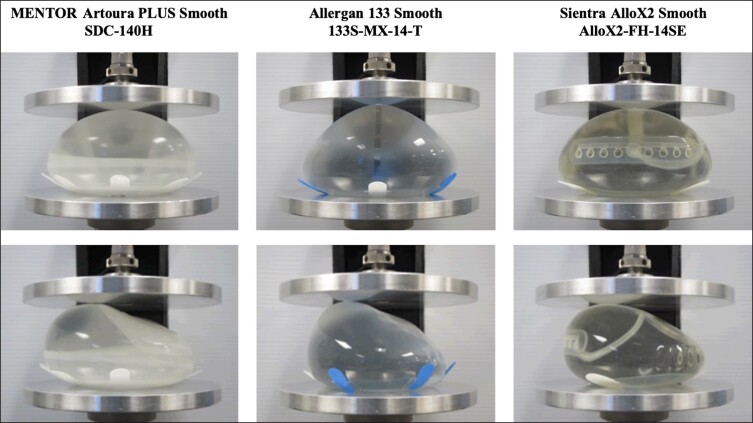
Breast tissue expanders shown pre-test (no load) at posterior (top panels) and lateral views (bottom panels). Products tested were MENTOR Artoura PLUS Smooth SDC-140H (Irvine, CA), Allergan 133 Smooth 133S-MX-14-T (Irvine, CA), and Sientra AlloX2 Smooth AlloX2-FH-14SE (Santa Barbara, CA).

Each TE was filled with exact volumes of room temperature liquid within ±3 cc of the target volume during testing. Baseline dimensions of width, height, and projection of each TE were recorded. Each expander was centered on the compression plates where a compressive load of 5 pounds was initially applied, and subsequent testing incrementally increased by 5-pound intervals up to 35 pounds (total range: 5-35 pounds). Figure 1 demonstrates the pre-test views and Supplemental Figure 1 shows pre-test views and compressed views at 35 pound-force (lbf) for the devices. Each TE experienced the compressive load until the expander’s width and height were calculated and recorded. A percent change of base height and width was calculated relative to baseline recordings and compressive measurements.

## RESULTS

### Mechanical Vertical Compression Testing

**Figure 2.**
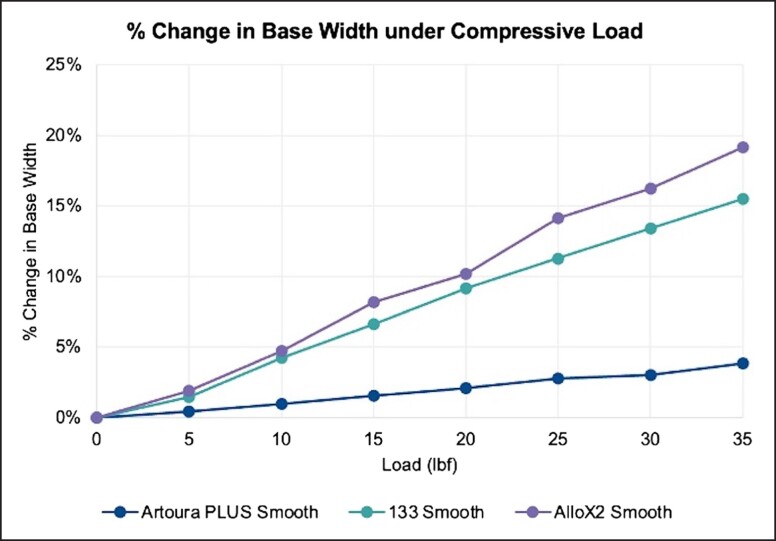
Change in base width under compressive loads at 100% fill volume. Products tested were Artoura PLUS Smooth (MENTOR, Irvine, CA), 133 Smooth (Allergan, Irvine, CA), and AlloX2 Smooth (Sientra, Santa Barbara, CA).

**Figure 3.**
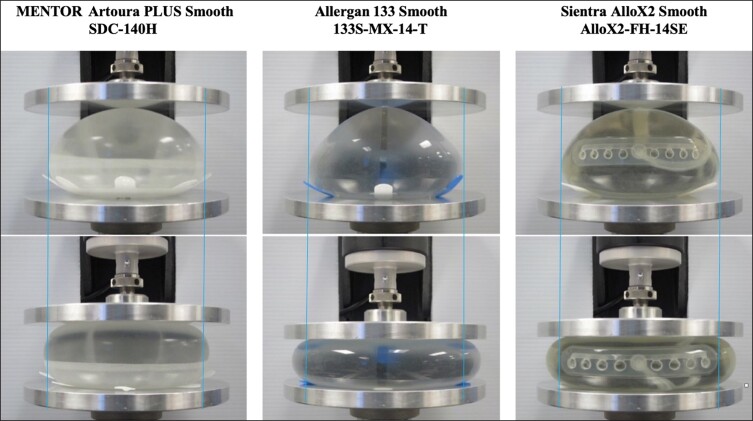
Top panels: device at pre-test (no load). Bottom panels: device at 35 pounds of compression (posterior view). Products tested were MENTOR Artoura PLUS Smooth SDC-140H (Irvine, CA), Allergan 133 Smooth 133S-MX-14-T (Irvine, CA), and Sientra AlloX2 Smooth AlloX2-FH-14SE (Santa Barbara, CA).

The summary of percent changes in base width under given loading parameters are reported in Figure 2 and Table 1. The MENTOR Artoura PLUS Smooth consistently demonstrated the least amount of lateral deformation over the range of experimental compressive loads compared with the other 2 devices. When compressive loads of 10, 20, and 35 pounds are applied, the MENTOR Artoura PLUS Smooth had a 0.98%, 2.09%, and 3.84% change in base width, respectively. In comparison, under the same compressive loads of 10, 20, and 35 pounds, the Allergan 133 Smooth experienced a 4.21%, 9.15%, and 15.52% change in base width and the Sientra AlloX2 Smooth experienced a 4.72%, 10.19%, and 19.15% change in base width, respectively. The trajectory of the device’s footprint under 35 pounds of compressive force is meant to simulate the physical load on a breast when a patient is lying face down or hugging.^9,10^ The physical pressure on the TE is documented by the displacement relative to its original starting width, indicated by the blue line in Figure 3.

**Table 1.** Change in Base Width Under Compressive Loads at 100% Fill Volume

Load (lbf)	Artoura PLUS Smooth SDC-140H (MENTOR, Irvine, CA)	133 Smooth 133S-MX-14-T (Allergan, Irvine, CA)	AlloX2 Smooth AlloX2-FH-14SE (Sientra, Santa Barbara, CA)
5	0.58	1.99	2.54
10	1.31	5.81	6.29
15	2.05	9.12	10.89
20	2.79	12.63	13.60
25	3.68	15.57	18.88
30	4.00	18.51	21.64
35	5.12	21.43	25.55

lbf, pound of force.

**Figure 4.**
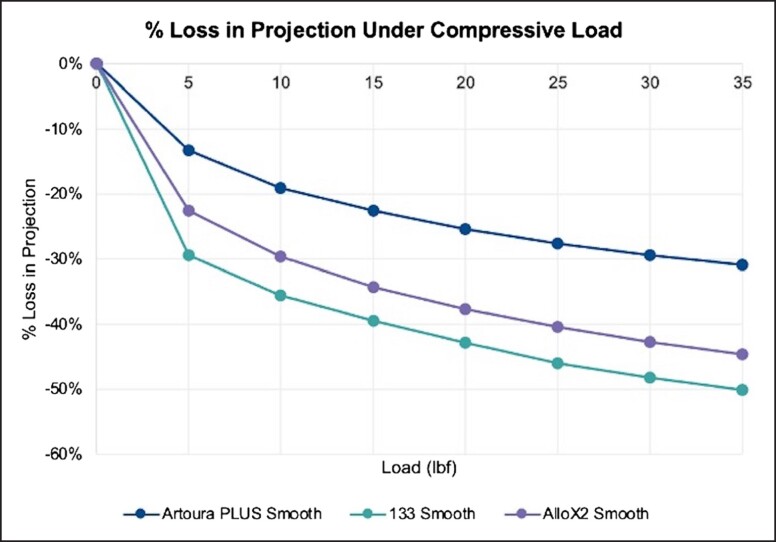
Loss in projection under compressive loads at 100% fill volume. Products tested were Artoura PLUS Smooth (MENTOR, Irvine, CA), 133 Smooth (Allergan, Irvine, CA), and AlloX2 Smooth (Sientra, Santa Barbara, CA).

The response to compressive loads on the device projection is reported in Figure 4 and Table 2. The MENTOR Artoura PLUS Smooth had the least amount of projection loss at each load that was tested. Testing at 10, 20, and 35 pounds of force yielded a −19.06%, −25.44%, and −30.88% change in projection for the MENTOR Artoura PLUS Smooth. In comparison, under the same compressive loads, the Allergan 133 Smooth experienced a −35.53%, −42.90%, and −50.09% change in projection and the Sientra AlloX2 Smooth experienced a −29.64%, −37.68%, and −44.69% change.

**Table 2.** Loss in Projection Under Compressive Loads at 100% Fill Volume

Load (lbf)	Artoura PLUS Smooth SDC-140H (MENTOR, Irvine, CA)	133 Smooth 133S-MX-14-T (Allergan, Irvine, CA)	AlloX2 Smooth AlloX2-FH-14SE (Sientra, Santa Barbara, CA)
5	−9.76	−22.04	−16.40
10	−13.96	−26.60	−21.51
15	−16.51	−29.52	−24.88
20	−18.64	−32.11	−27.34
25	−20.25	−34.43	−29.32
30	−21.58	−36.11	−30.99
35	−22.62	−37.50	−32.42

lbf, pound of force.

**Figure 5.**
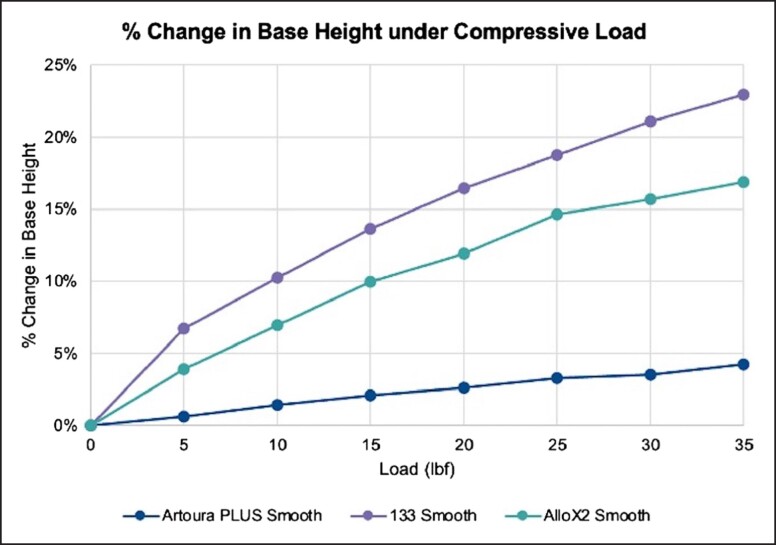
Change in base height under compressive loads at 100% fill volume. Products tested were Artoura PLUS Smooth (MENTOR, Irvine, CA), 133 Smooth (Allergan, Irvine, CA), and AlloX2 Smooth (Sientra, Santa Barbara, CA).

The change in height under loaded conditions is reported in Figure 5 and Table 3. The MENTOR Artoura PLUS Smooth had the least change in height at each of the tested loads. Testing at 10, 20, and 35 pounds of force yielded a 1.44%, 2.62%, and 4.27% change in height for the MENTOR Artoura PLUS Smooth. In comparison, under the same compressive loads, the Allergan 133 Smooth experienced a 10.26%, 16.49%, and 22.97% change in height and the Sientra AlloX2 Smooth experienced a 6.99%, 11.93%, and 16.90% change.

**Table 3.** Change in Base Height Under Compressive Loads at 100% Fill Volume

Load (lbf)	Artoura PLUS Smooth SDC-140H (MENTOR, Irvine, CA)	133 Smooth 133S-MX-14-T (Allergan, Irvine, CA)	AlloX2 Smooth AlloX2-FH-14SE (Sientra, Santa Barbara, CA)
5	0.81	8.34	4.92
10	1.93	12.70	8.76
15	2.82	16.91	12.49
20	3.50	20.41	14.96
25	4.37	23.24	18.39
30	4.73	26.09	19.69
35	5.70	28.43	21.19

lbf, pound of force.

Each breast TE's ability to resist external compressive forces, a marker for distortion and control over tissue expansion, was evaluated with results summarized in Supplemental Table 2. The distance the top plate traveled from pre-testing vs once a 35-pound pressure was applied can be seen in Supplemental Figure 2. The distance the top plate traveled was the lowest in the MENTOR Artoura PLUS Smooth, followed by the Sientra AlloX2 Smooth and the Allergan 133 Smooth.

## DISCUSSION

Immediate breast reconstruction following mastectomy has continued to increase in popularity, partly facilitated by the adoption of the alloplastic approach.^1,11,12^ Using a temporary TE for breast pocket creation, the strategy expands the scope of aesthetic outcomes and therapeutic flexibility by counteracting the effects of adjuvant radiotherapy. However, delivery of optimal aesthetic and functional outcomes is partially contingent upon a thorough understanding and predictability of TE behavior under physiologic scenarios.^4,13,14^

Unfortunately, TEs have limitations, including unwanted lateral distortion of the base and attenuated forward projection with physiologic compressive forces of the chest wall and soft tissues.^4,15^ These unknown changes result in diminished accuracy in surgical planning and necessitate additional corrective surgical procedures to form an ideal breast pocket.^4,13,16^

Our benchtop analysis seeks to characterize the dynamic behavior and variability among TEs. Equipped with a thorough understanding of the device, a reconstructive surgeon can better select the most appropriate device to achieve intended results and minimize complications.^17,18^

In this study, 3 commercially available TEs were directly compared. Our goal was to adequately capture static physiologic loads and intermittent stresses such as lying flat on the device. Mechanical vertical compression testing assessed changes in base width, height, and lateral projection. The MENTOR Artoura PLUS Smooth consistently demonstrated the least amount of lateral base deformation, loss of projection, and greatest ability to withstand external compressive forces. While the Allergan 133 Smooth placed second for lateral deformation, it was third for loss of projection and ability to resist compressive forces when compared with the Sientra AlloX2 Smooth.

A crucial consideration for surgeons and patients when selecting TEs for staged breast reconstruction is the ability of the TE to maintain its contour under the pressure of an external force.^19,20^ When the MENTOR Artoura PLUS Smooth, Allergan 133 Smooth, and Sientra AlloX2 Smooth TEs were placed under compressive loads ranging from 5 to 35 pounds, the MENTOR Artoura PLUS Smooth showed the least amount of lateral deformation across all of the compressive loads. For surgeons, minimizing lateral deformation of TEs is essential to improve our ability to accurately select an implant that matches the dimensions desired and decreases the need for capsulorrhaphy during the second stages of breast reconstruction.^21^ We recommend that surgeons should be cognizant of TEs that have greater lateral distortion profiles and select a wider implant during the second stage to account for the greater expansion in width. The precision of the TE is beneficial since there are many factors outside of the surgeon’s control that can impact the outcomes of subpectoral reconstruction, such as the degree of mastectomy dissection, destruction of the lateral breast footprint and inframammary fold, patient movement after the device is implanted, the effects of radiation therapy on the elasticity of the tissue, and gravity or muscle contraction forces acting on the TE.^22^ Overall, the use of a TE with minimal lateral deformation reduces operation time, decreasing the probability of complications such as bleeding and infection.^16,23,24^ A device that is able to maintain its footprint or projection when subjected to compressive loads may offer advantages clinically over the long-term.

The effect of compressive loads on the device projection of TEs is significant to patients as they impact functional and cosmetic outcomes.^13^ Testing demonstrated that the MENTOR Artoura PLUS Smooth had the least amount of projection loss at each respective load compared with its other TE counterparts. Distortion and control over tissue expansion can be quantified through the TE's ability to resist external compressive forces. The MENTOR Artoura PLUS Smooth breast TE was able to resist compressive forces the most, followed by the Sientra AlloX2 Smooth and the Allergan 133 Smooth. TEs that can maintain their projection throughout daily patient activities such as hugging, lying prone, and during other movements and positions are more likely to result in the natural teardrop shape of the breast and reduce the risk for complications or subsequent surgeries.^25,26^

### Limitations

While this study allowed for a practical simulation of pressure applied to the TEs between the skin and the chest wall, there are limitations to the study design. First, the compressive force used in this study was very uniform and unidirectional, while real-life activities can distort the TE via localized points of pressure and not only in the anteroposterior direction. Due to the study technique used in this investigation, additional factors known to impact breast reconstruction, such as radiation, body mass index, age, the extent of dissection, and comorbidities, were not analyzed. Therefore, the confounding variables that can alter the compression exerted on a TE, specifically body mass index, the thickness of the mastectomy flaps, and radiation-induced skin fibrosis, were not examined. This study cannot imitate the lateral migration of the TE as the degree of lateral dissection during the mastectomy can be variable between patients. The study's goal was not to push the devices to their maximum failure point, but it would have been valuable to know the ultimate weight that each TE could support. Additionally, this simulation does not account for longitudinal changes in TE over days, weeks, and months. Similar clinical testing can be performed on patients after Institutional Review Board approval to further build upon the results of this study. Analyzing TEs in vivo via quantifiable metrics would allow for comparing distortions within the soft-tissue envelope and create a more realistic representation of multi-directional pressure vectors.

We chose to investigate 3 common brands of TEs utilized at our institution to provide surgeons with additional data points when making an informed choice for their patients. This study has several strengths in its study design and comprehensive testing framework comparing 3 popular commercially available TEs. Hopefully, this study can serve as an outline of how to directly compare TEs, especially as new devices enter the market.

## CONCLUSIONS

Our analysis found that the MENTOR Artoura PLUS Smooth was superior in minimizing lateral displacement and maintaining the lower pole projection observed during testing. This knowledge offers information to determine dimensions for the second stage of reconstruction based on the surgeon's reconstructive goals. The framework herein provides a method to compare breast TEs against each other and determine a mechanical profile for each device to improve aesthetic outcomes. Future studies should focus on additional mechanical properties that can be explored to help surgical planning and patient outcomes.

## Supplementary Material

ojad018_Supplementary_Data
