# Supplementary material for: The Best Under Stress: An Analysis of Breast Tissue Expander Response to External Forces
Source: Aesthet Surg J Open Forum. 2023 Feb 20;5:ojad018. doi: 10.1093/asjof/ojad018 (PMC10063436; doi:10.1093/asjof/ojad018)
Supplement: ojad018_Supplementary_Data [file ojad018_supplementary_data.zip › 22-0113_Supplemental Table 2.docx]

**Supplemental Table 2**: Results Summary at 100% Fill Volume for Change Under Load and Percent Change Under Load

| Device  SKU | Label vol. (cc) | Baseline – no load | | | Load (lbf) | Change under load | | | % change under load | | |
| --- | --- | --- | --- | --- | --- | --- | --- | --- | --- | --- | --- |
|  |  | Width (mm) | Height (mm) | Proj. (mm) |  | Width (mm) | Height (mm) | Proj. (mm) | Width (%) | Height (%) | Proj. (%) |
| Artoura PLUS Smooth  SDC-140H (MENTOR, Irvine, CA) | 600 | 133.47 | 133.61 | 73.25 | 5 | 0.58 | 0.81 | -9.76 | 0.43% | 0.61% | -13.32% |
|  |  |  |  |  | 10 | 1.31 | 1.93 | -13.96 | 0.98% | 1.44% | -19.06% |
|  |  |  |  |  | 15 | 2.05 | 2.82 | -16.51 | 1.54% | 2.11% | -22.54% |
|  |  |  |  |  | 20 | 2.79 | 3.50 | -18.64 | 2.09% | 2.62% | -25.44% |
|  |  |  |  |  | 25 | 3.68 | 4.37 | -20.25 | 2.76% | 3.27% | -27.64% |
|  |  |  |  |  | 30 | 4.00 | 4.73 | -21.58 | 3.00% | 3.54% | -29.46% |
|  |  |  |  |  | 35 | 5.12 | 5.70 | -22.62 | 3.84% | 4.27% | -30.88% |
| 133 Smooth  133S-MX-14-T (Allergan, Irvine, CA) | 600 | 138.07 | 123.76 | 74.86 | 5 | 1.99 | 8.34 | -22.04 | 1.45% | 6.74% | -29.44% |
|  |  |  |  |  | 10 | 5.81 | 12.70 | -26.60 | 4.21% | 10.26% | -35.53% |
|  |  |  |  |  | 15 | 9.12 | 16.91 | -29.52 | 6.61% | 13.67% | -39.43% |
|  |  |  |  |  | 20 | 12.63 | 20.41 | -32.11 | 9.15% | 16.49% | -42.90% |
|  |  |  |  |  | 25 | 15.57 | 23.24 | -34.43 | 11.28% | 18.78% | -45.99% |
|  |  |  |  |  | 30 | 18.51 | 26.09 | -36.11 | 13.40% | 21.08% | -48.24% |
|  |  |  |  |  | 35 | 21.43 | 28.43 | -37.50 | 15.52% | 22.97% | -50.09% |
| AlloX2 Smooth  AlloX2-FH-14SE (Sientra, Santa Barbara, CA) | 575 | 133.39 | 125.32 | 72.55 | 5 | 2.54 | 4.92 | -16.40 | 1.90% | 3.92% | -22.61% |
|  |  |  |  |  | 10 | 6.29 | 8.76 | -21.51 | 4.72% | 6.99% | -29.64% |
|  |  |  |  |  | 15 | 10.89 | 12.49 | -24.88 | 8.16% | 9.96% | -34.29% |
|  |  |  |  |  | 20 | 13.60 | 14.96 | -27.34 | 10.19% | 11.93% | -37.68% |
|  |  |  |  |  | 25 | 18.88 | 18.39 | -29.32 | 14.15% | 14.67% | -40.42% |
|  |  |  |  |  | 30 | 21.64 | 19.69 | -30.99 | 16.22% | 15.71% | -42.72% |
|  |  |  |  |  | 35 | 25.55 | 21.19 | -32.42 | 19.15% | 16.90% | -44.69% |

cc, cubic centimeter; lbf, pound of force; mm, millimeter; SKU, stock keeping unit.
