# Supplementary material for: The Best Under Stress: An Analysis of Breast Tissue Expander Response to External Forces
Source: Aesthet Surg J Open Forum. 2023 Feb 20;5:ojad018. doi: 10.1093/asjof/ojad018 (PMC10063436; doi:10.1093/asjof/ojad018)
Supplement: ojad018_Supplementary_Data [file ojad018_supplementary_data.zip › 22-0113_Supplemental Table 1.docx]

**Supplemental Table 1**: Summary of Manufacturer Specifications and Tissue Expanders Used in Vertical Compression Testing

| Manufacturer | Product family | Style | Product code | Label volume and dimensions | | | |
| --- | --- | --- | --- | --- | --- | --- | --- |
|  |  |  |  | Volume (cc) | Width (cm) | Height (cm) | Projection (cm) |
| MENTOR (Irvine, CA) | Artoura PLUS Smooth | High profile | SDC-140H | 600 | 14.0 | 14.0 | 7.1 |
|  |  |  |  |  |  |  |  |
|  |  |  |  |  |  |  |  |
| Allergan (Irvine, CA) | 133 Smooth | Moderate height | 133S-MX-14-T | 600 | 14.0 | 13.0 | 7.1 |
|  |  |  |  |  |  |  |  |
|  |  |  |  |  |  |  |  |
| Sientra (Santa Barbara, CA) | AlloX2 Smooth | Full height | AlloX2-FH-14SE | 480 - 575 | 14.0 | 12.9 | 6.1 - 7.3 |

cc, cubic centimeter; cm, centimeters.
