# Supplementary figures and images for: The Best Under Stress: An Analysis of Breast Tissue Expander Response to External Forces
Source: Aesthet Surg J Open Forum. 2023 Feb 20;5:ojad018. doi: 10.1093/asjof/ojad018 (PMC10063436; doi:10.1093/asjof/ojad018)

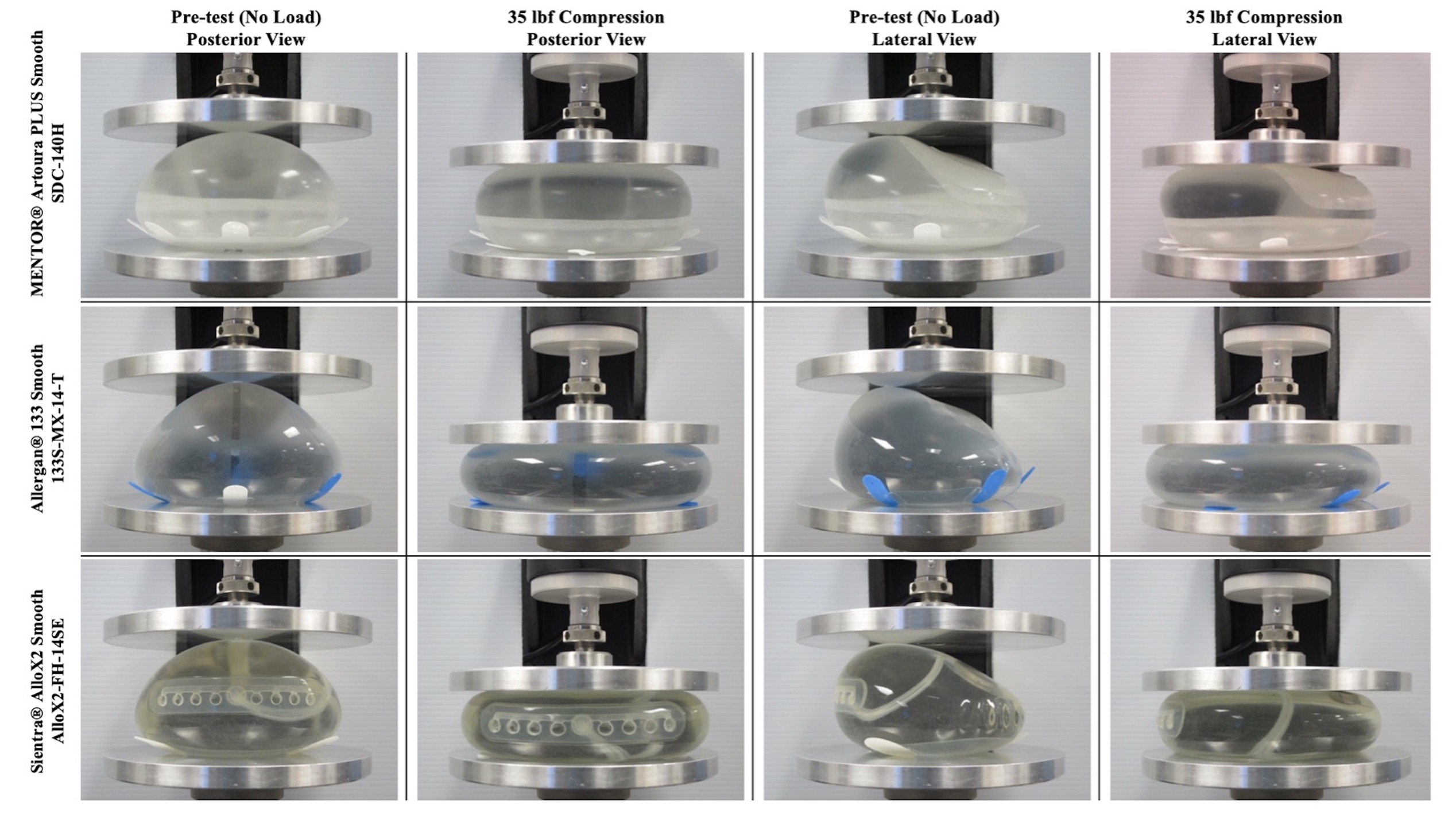

Supplement: ojad018_Supplementary_Data [file ojad018_supplementary_data.zip › Supplemental Figure 1.jpg]

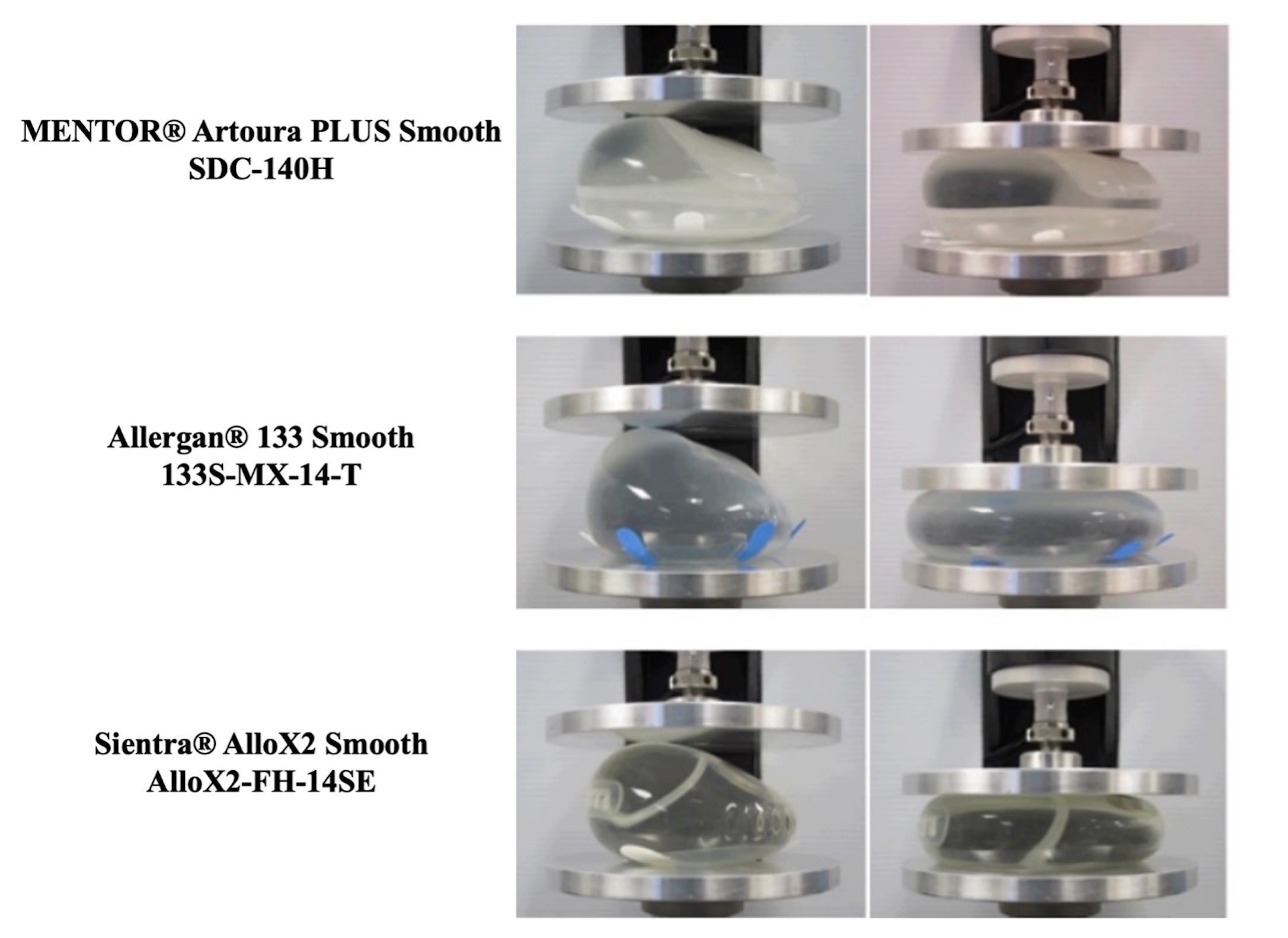

Supplement: ojad018_Supplementary_Data [file ojad018_supplementary_data.zip › 22-0113_Supplemental Figure 2.jpg]
